# Reply to Harwood et al.: Alternative functional conformations of native human α_2_-macroglobulin

**DOI:** 10.1073/pnas.2211048119

**Published:** 2022-08-16

**Authors:** Daniel Luque, Theodoros Goulas, Carlos P. Mata, Soraia R. Mendes, F. Xavier Gomis-Rüth, José R. Castón

**Affiliations:** ^a^Spanish National Microbiology Centre, Institute of Health Carlos III, 28220 Madrid, Spain;; ^b^Proteolysis Lab, Department of Structural Biology, Molecular Biology Institute of Barcelona, Higher Scientific Research Council (CSIC), Barcelona, 08028 Catalonia, Spain;; ^c^Department of Food Science and Nutrition, School of Agricultural Sciences, University of Thessaly, 43100 Karditsa, Greece;; ^d^Astbury Centre for Structural Molecular Biology, School of Molecular and Cellular Biology, Faculty of Biological Sciences, University of Leeds, Leeds LS2 9JT, United Kingdom;; ^e^Department of Structure of Macromolecules, Centro Nacional de Biotecnología, Consejo Superior de Investigaciones Científicas, 28049 Madrid, Spain

Harwood et al. (1) challenge our study of human α_2_-macroglobulin (hα_2_M) by cryogenic electron microscopy (2) based on differences with other α_2_M-family members. This discrepancy would result from damaged protein caused by the usage of thawed frozen fresh plasma (TFP) for protein preparation. Instead, “homogeneously native” hα_2_M would be purified from fresh, nonfrozen plasma (NP).

To address this issue, we compared hα_2_M purified from TFP and NP. Briefly, we found that both preparations were equivalent in electrophoretic migration patterns, ultraviolet absorbance profiles, molar mass distributions, and free thiol groups. No differences in the inhibitory capacity against trypsin and thermolysin with three different substrates were detected. Thus, the preparations were equivalent, active, and functional, as already mentioned in our paper (2). This confirms reports from many groups which have been using frozen fresh plasma over decades (3–6). In particular, ref. 7 reckoned that frozen plasma may be used for native hα_2_M preparation.

Recently, a comparative study with NP samples was published by Huang et al. (8), which shows that native preparations contain particles with multiple conformations (figure 2 of ref. 8). This is in agreement with very early studies (9). Thus, purified, native hα_2_M from NP does not evince a unique structure as postulated by Harwood et al. (1) but rather a compendium of them, as we found from TFP. In ref. 2, we performed in silico separation and analysis of several subclasses of particles (see figures S3 and S9 and table S1 in ref. 2). Specifically, ∼46,000 images were included in the native I set, which rules out that the protein was randomly damaged (tables S1 and S4 of ref. 2). In addition, the overall conformation of the fully native tetramer of ref. 8 is very similar to ours (compare figure 1B of ref. 8 with figures 2 and 3 of ref. 2). In particular, the MG7–CUB(TED)–RBD block adopts a similar overall conformation in both “extended” (2) and “expanded” (8) protomers, in which the thioester bond is protected by a hydrophobic circus and the nearby presence of RBD (see figure 4 E and F of ref. 2). There just seems to be a relative rearrangement of the RBDs.

As to native α_2_M-family members, the mentioned proteins operate as monomers through completely different mechanisms, except frog ovostatin, which occurs in a physiologically different context from hα_2_M. In addition, α_2_M-family members evince substantially different activated structures. Thus, consistent with disparate functions, there is no structural or functional homogeneity. However, we cannot exclude that the extended protomer of hα_2_M may sample alternative conformations while maintaining a functional tetrameric structure. This is consistent with the large flexibility and conformational disparity found in the theoretically homogeneous native preparations. Note also that this variability (figure S2 of ref. 2) is reduced to a single activated tetramer conformation upon treatment with trypsin (figure S4 of ref. 2), which further underpins that our native preparations are functionally competent.

Overall, the claims that our structures be artifacts resulting from damaged samples are not substantiated. Our study (2) and that of Huang et al. (8) complementarily recapitulate the mechanism of function of hα_2_M.
